# Reductant free green synthesis of magnetically recyclable MnFe_2_O_4_@SiO_2_-Ag coreshell nanocatalyst for the direct reduction of organic dye pollutants

**DOI:** 10.3906/kim-2108-2

**Published:** 2021-09-20

**Authors:** Ali Serol ERTÜRK, Gökhan ELMACI, Mustafa Ulvi GÜRBÜZ

**Affiliations:** 1 Department of Analytical Chemistry, Faculty of Pharmacy, Adıyaman University, Adıyaman, Turkey; 2 Department of Chemistry, School of Technical Sciences, Adıyaman University, Adıyaman, Turkey; 3 Department of Chemistry, Faculty of Arts and Sciences, Yıldız Technical University, İstanbul, Turkey

**Keywords:** Magnetic recyclable nanocatalyst, *Epilobium parviflorum*, silver nanoparticle, heterogeneous catalyst, reduction of organic dyes

## Abstract

The present paper describes *in situ* green immobilization of silver nanoparticles on MnFe_2_O_4_@SiO_2_ nanospheres using *Epilobium parviflorum* (*EP*) without using any other toxic chemicals and reducing or stabilizing agents. The morphology, composition, and magnetic properties of the resulting MnFe_2_O_4_@SiO_2_-Ag core-shell nanocatalyst were characterized by scanning electron microscope (SEM), transmission electron microscopy (TEM), thermogravimetric analysis (TGA), X-ray diffraction (XRD), vibrating sample magnetometer (VSM), and attenuated total reflectance-Fourier transform infrared spectroscopy (ATR-FTIR). The catalytic performance of the synthesized MnFe_2_O_4_@SiO_2_-Ag was employed on the organic pollutants dyes such as rhodamine B (RhB) and methylene blue (MB). The results revealed significant reduction performances for the MB (116.28 s^−1^ g^−1^) and RhB (27.12 s^−1^ g^−1^) over the existing literature. Furthermore, the MnFe_2_O_4_@SiO_2_-Ag exhibited high stability for the completion of the reduction of RhB between the reaction times of 13.1 (first) and 19.8 min (final) with the 100% decolorization efficiency even after several cycles with an excellent magnetic separation. Overall, this work demonstrates a simple and practical green synthetic route for the preparation of magnetic recyclable core-shell nanocatalyst that can be a good candidate for the treatment of organic contaminants in wastewater adhering to green chemistry principles for the environmental pollution concerns.

## 1. Introduction

Organic dye contaminants have become an acute concern and problem in the environment due to their release or discharge into the environment as arising intensive activities of different chemical industries, including food, textile, cosmetics, plastics, paint, and indeed domestic waste [1,2]. Most of these waste dyestuffs or effluents are toxic, carcinogenic, and even mutagenic, as well as posing serious risks to living organisms, especially to human health [3–6]. Although diverse techniques involving adsorption, precipitation, photocatalytic degradation, and advanced oxidation processes (AOPs) have been introduced to treat organic dye pollutants up to now, they could be most frequently time-consuming, impractical, and expensive [7–9]. For these reasons, there has been still a growing interest to develop methods or strategies for the removal of dye pollutants before their release from various industries into the environment.

Based on this purpose, metal nanoparticles with higher Fermi potential that enable them to catalyze electron transfer reaction with lowered reduction potential have attracted great interest in reducing organic dye pollutants [10,11]. In particular, silver nanoparticles (AgNPs) among several noble metal-based catalysts containing gold, palladium, and platinum have gained significant research and application for a variety of catalytic reactions, some of which are reduction of organic compounds, selective oxidation, and NO_x_ reduction, because of their unique properties, including low-cost, high optical, catalytic, and antibacterial properties [12,13]. In this point, not only the use of reducing agents in the production of AgNPs might lead to environmental toxicity and biohazards but also because the industry promotes catalytic processes with ease operation, employ and recyclability, the use of green synthetic roots and environment in preparing a heterogeneous catalyst remains among the main research principles [14,15]. For this reason, magnetic nanoparticles (MNPs) have received much interest in the heterogeneous catalyst as a useful support owing to their ease of separation from the reaction media using an external magnetic field compared to filtration and centrifugation processes, high dispersion, and recyclability [16–18]. Therefore, MNPs can improve the separation and recovery of AgNPs from the reaction media.

Among different coating materials, silica as a protective shell can be facilitated to maintain the stability of MNPs and prevent their interaction with complex matrices with the desired stability [19,20]. In addition, plant-mediated synthesis of nanoparticles has attracted great attention depending on its several advantages, comprising non-toxic, safe, cost effective, especially being environmentally friendly [21–24]. Thus, the aforementioned environmental concerns can be overcome in the fast and economic production of magnetic core-shell nanoparticles with more stable properties via the immobilization of silver nanoparticles on silica coated MNPs by using plant extracts as reducing agents.

In our previous study, we have introduced *Epilobium parviflorum* (*EP*) extract as a novel reducing, stabilizing agent, and coating material for the preparation of Ag immobilized nanocatalyst using manganese ferrite magnetic core as an alternative to commonly used Fe_3_O_4_ core supports [25]. Apart from this study, we addressed herein the green and successful preparation technique for the synthesis of highly stable MnFe_2_O_4_@SiO_2_-Ag core-shell magnetically recyclable nanocatalyst using *EP* extract for the first time. In this perspective, the current research has come to a focal point as the used *EP* extracts serve on the basis of the green synthesis of heterogeneous catalyst without using any additional chemicals, stabilizer, surfactant, toxic or extra reducing agents, and become inspiring for the future studied dealing with more environmental concerns. The MnFe_2_O_4_@SiO_2_-Ag has also been investigated as a useful catalyst in the reduction of some organic pollutant dyes.

## 2. Materials and methods

### 2.1. Chemicals and materials

Iron (III) chloride hexahydrate (FeCl_3_.6H_2_O), manganese (II) chloride tetrahydrate (MnCl_2_.4H_2_O), ammonia (NH_3_), silver nitrate (AgNO_3_), polyvinylpyrrolidone (PVP), methylene blue (MB), and rhodamine B (RhB) were purchased from Sigma-Aldrich and used without any further purifications. *Epilobium parviflorum* (EP) plant (green tea extract) was purchased from the local market in Turkey.

### 2.2. Instrumentation

A Pan Analytical Empyrean diffractometer with a PixCell3D detector was used for the Powder X-ray diffraction pattern (XRD) measurements. Attenuated total reflectance-Fourier transform infrared spectroscopy (ATR-FTIR) spectra were collected between the wavelength range of 600–4000 cm^−1^ via PerkinElmer Spectrum 100 FT-IR Spectrometer. The water content of the samples was detected using TA Instrument (New Castle, DE) thermal analysis system with a heating program of 10 °C min^−1^ under air flow (100 mL min^−1^) by thermogravimetry. The morphological analyses were carried out using an electron microscope (SEM, ZEISS Sigma 300) integrated with energy-dispersive X-ray spectroscopy (EDS), and high contrast transmission electron microscope (TEM, Hitachi HT7700 with EXALENS). UV-Vis measurements were carried out via a Carry 60 UV-Vis spectrometer, (Agilent, USA) with a 1 cm quartz cell.

### 2.3. Synthesis of MnFe_2_O_4_ nanoparticles

A mixture of Iron (III) chloride hexahydrate (FeCl_3_.6H_2_O) (6.5 g) and manganese (II) chloride tetrahydrate (MnCl_2_.4H_2_O) (4.0 g), and 0.2 g polyvinylpyrrolidone (PVP) in 80 mL of de-ionized water (100 mL) was stirred vigorously for 3 h. Afterwards, 20 mL of 0.1M NH_4_OH solution was slowly added and irradiated under microwave for 20 min at 100 °C. After cooling the reaction mixture to room temperature, the black precipitate of MnFe_2_O_4_ nanoparticles were separated magnetically and washed three times with mixture of ethanol-deionized water [25].

### 2.4. Synthesis of MnFe_2_O_4_@SiO_2_ core-shell nanoparticles

Synthesis of MnFe_2_O_4_@SiO_2_ core-shell nanoparticles were simply adopted from the literature [20]. In summary, 1.0 g of MnFe_2_O_4_ nanoparticles were added to a solution of 5.0 mL of NH_4_OH (25%) and 200.0 mL of ethanol and dispersed well. 2.5 mL of tetraethyl orthosilicate was added over the resulting mixture dropwise while vigorously stirring. After stirring the mixture for 12 h at 40 °C, the obtained MnFe_2_O_4_@SiO_2_ nanoparticles were separated using an external magnet, washed several times with ethanol, and dried at room temperature.

### 2.5. Synthesis of MnFe_2_O_4_@SiO_2_ -Ag

The preparation of *EP* green tea extract was reported in our recent study [26]. For further synthesis of the MnFe_2_O_4_@SiO_2_-Ag nanocatalyst, 50 mg of MnFe_2_O_4_@SiO_2_ was added over a stirring solution of 50.0 mL of AgNO_3_ (0.15 mM) and dispersed well. Afterwards, 8.0 mL of the *EP* extract was added while constant stirring at 50 °C for 60 min. After cooling the reaction mixture to room temperature, the precipitates were collected by using a niobium magnet and washed several times with distilled water and, later on, with three times with ethanol to get rid of impurities [20].

### 2.6. Catalytic activity of MnFe_2_O_4_@SiO_2_-Ag

The catalytic performance of MnFe_2_O_4_@SiO_2_-Ag was tested over the reduction reaction of RhB and MB by NaBH_4_. Prior to the catalytic reactions, in order to completely achieve adsorption-desorption equilibrium, the MnFe_2_O_4_@SiO_2_-Ag NPs (20 μL, 2.15 mg mL^−1^), de-ionized water (0.75 mL), and RhB (40 μL, 3.06 mM) were stirred for 30 min. After that, 2.25 mL portion of the 0.1 M NaBH_4_ was poured into this solution. By adopting the same procedure, the catalytic assays were completed by using MB (10 μL, 2.25 mM) and NaBH_4_ (2.25 mL, 0.1 M). UV-Vis measurements were recorded between the range of 350–700 nm and 500–750 nm for RhB ((λ_max_ = 554 nm) and MB (λ_max_ = 670 nm) to monitor the performed reaction until bleaching the color of the aqueous solutions of dyes.

## 3. Results and discussion

### 3.1. Synthesis and characterization

**Scheme 1:**
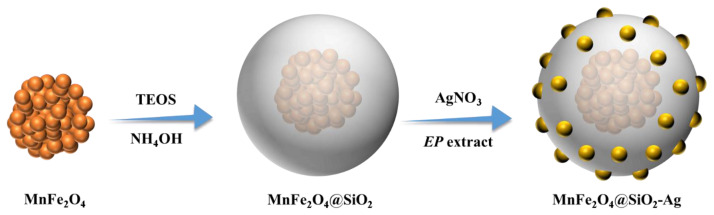
Systematic synthetic route for the production of MnFe_2_O_4_@SiO_2_-Ag nanocatalyst.

In the nanocomposite catalyst design, MnFe_2_O_4_ was chosen as it has high saturation magnetization value and rough surface [17]. Then, the MnFe_2_O_4_ surface was coated with SiO_2_ thin layer to prevent agglomeration and create a porous area [27]. The facility of the *EP* green tea extract for the reduction and stabilization of metal nanoparticles as coating material with its rich content in terms of phenolic compound derivatives such as tannins, flavonoids, and phenolic acids has been demonstrated in our recent study [25]. Considering this potential of *EP* extracts, herein, we employed them as efficient reducing agents for the immobilization of AgNPs on the protective SiO_2_ outer layer. Therefore, the resulting MnFe_2_O_4_@SiO_2_-Ag nanocomposite can be used as a low-cost, recyclable, environmentally friendly, and active catalyst platform. The experimental strategy for the preparation of MnFe_2_O_4_@SiO_2_-Ag was illustrated in Scheme 1. The MnFe_2_O_4_@SiO_2_-Ag was synthesized in two-step approach. In the first step, the silica layer was coated on the magnetic core nanoparticle, MnFe_2_O_4_. In the next step, silver ions was adsorbed and *in situ* reduced on the surface of the MnFe_2_O_4_@SiO_2_ core-shell nanospheres by means of *EP* green tea extract in aqueous solution without using any other organic solvent, stabilizing, or reducing agents.

**Figure 1:**
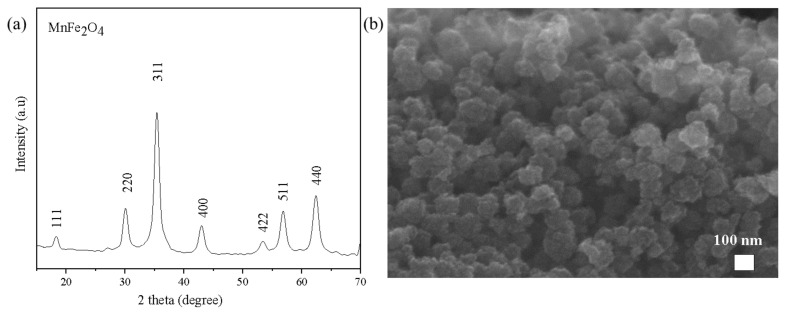
X-ray diffraction pattern (a) and SEM image of MnFe_2_O_4_ (b).

The crystalline phase, morphology, and particle size of the as prepared MnFe_2_O_4_@SiO_2_-Ag samples were examined via X-ray diffraction (XRD), Scanning Electron Microscopy (SEM), and Transmission Electron Microscopy (TEM). Figure 1 a shows the XRD patterns of MnFe_2_O_4_ nanoparticles. The spinel MnFe_2_O_4_ displayed peaks at 2θ values of 18.3° (111), 30.2° (220), 35.5° (311), 43.1° (400), 53.5° (422), 57.1° (511), and 62.6° (440), which can be indexed to the JCPDS 17–465 [28,29]. The SEM image of MnFe_2_O_4_ shows aggregates of well-defined spherical-like particles of sizes between 100–150 nm (Figure 1b).

**Figure 2:**
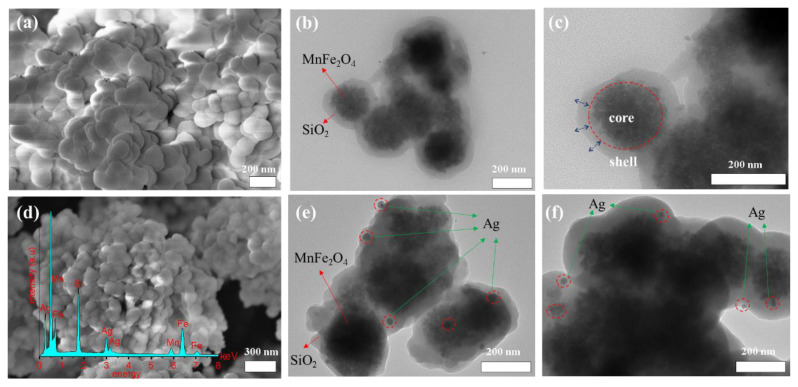
SEM image of MnFe_2_O_4_@SiO_2_ core-shell NPs (a). TEM image of MnFe_2_O_4_@SiO_2_ core-shell NPs (b, c). SEM image and Energy Dispersive Spectroscopy (EDS) analysis of MnFe_2_O_4_@SiO_2_-Ag core-shell NPs (d). TEM image of MnFe_2_O_4_@SiO_2_-Ag core-shell NPs (e, f).

The SEM micrographs of MnFe_2_O_4_@SiO_2_ and MnFe_2_O_4_@SiO_2_-Ag core-shell NPs are shown in Figures 2a–2d. In the current study, SiO_2_ thin layer was coated on the surface of MnFe_2_O_4_ magnetic core by hydrolysis of TEOS [30,31]. The SEM image of MnFe_2_O_4_@SiO_2_ showed that the MnFe_2_O_4_ core was homogeneously and successfully coated with SiO_2_ layer. The detailed core-shell structure was further confirmed by high-resolution TEM image (Figure 2b, 2c) and EDS (Figure 2d). It can be seen from Figure 2e that an amorphous SiO_2_ layer with a thickness of ~20 nm was homogeneously distributed over the surface of MnFe_2_O_4_. Moreover, The EDS analysis of MnFe_2_O_4_@SiO_2_-Ag clearly displayed signals from Ag, Mn, Fe, and Si atoms (Figure 2d). In this point, our previous study confirms that AgNPs are formed as a result of the *in situ* upon oxidation of active phenolic functional groups and derivatives in the *EP* extract by Ag^+^ ions at neutral pH value [25]. The resulting AgNPs were observed in spherical shape with 15 nm of average particle size in TEM analysis (Figure 2e, 2f). These results suggest that the AgNPs could be formed in every layer of the SiO_2_ layer. Thus, porous outer shell coated on the magnetic support can create a platform for the acceleration of mass-energy transfer to active catalysts such as Ag, Au, Pd, etc. [32,33].

**Figure 3:**
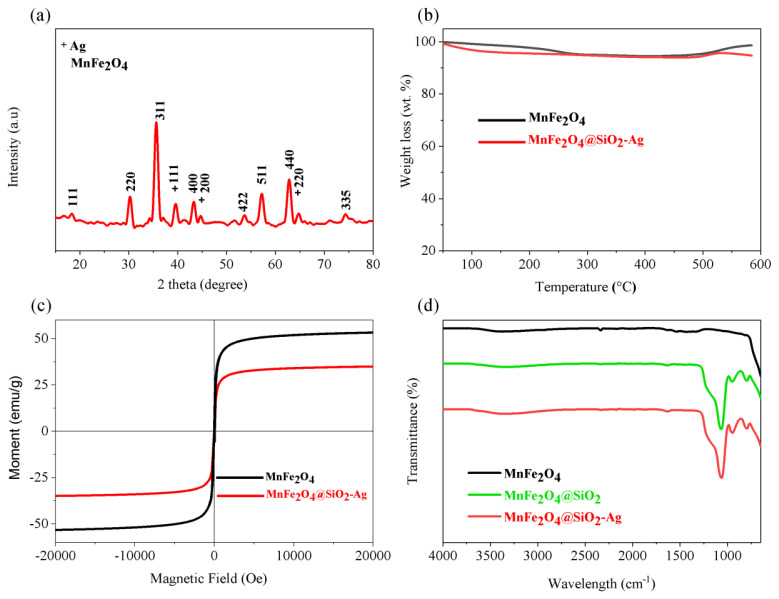
X-ray diffraction pattern of MnFe_2_O_4_@SiO_2_-Ag (a), TGA curves of MnFe_2_O_4_ and MnFe_2_O_4_@SiO_2_-Ag (b), Magnetic curves of MnFe_2_O_4_ and MnFe_2_O_4_@SiO_2_-Ag (c), ATR-FTIR spectra of MnFe_2_O_4_ and MnFe_2_O_4_@SiO_2_-Ag (d).

XRD patterns of the MnFe_2_O_4_@SiO_2_-Ag contain peaks of both crystalline MnFe_2_O_4_ and AgNPs (Figure 3a). The sharp diffraction peaks at 2θ = 38.2°, 44.3°, 64.5° and 76° can be indexed to the reflections of the (111), (200) and (220) crystalline planes of face-centered-cubic Ag (JCPDS card no. 04-0783), respectively [25]. In order to further confirm the composition and structure of the MnFe_2_O_4_@SiO_2_-Ag, thermal stability was investigated. A mass loss of MnFe_2_O_4_ is 5% up to 280 °C due to the volatilization of physically absorbed water and residual organic surfactant. As for MnFe_2_O_4_@SiO_2_-Ag, the mass loss is 1% higher than that of MnFe_2_O_4_ due to the decomposition of the thin layer of SiO_2_ [34] (Figure 3b).

The low recovery costs of catalysts are a significant factor in the development of sustainable catalyst systems [35–41]. Therefore, magnetically supported catalyst systems are considered to be one of the most important platforms as they can be easily separated from the reaction media via the aid of an external magnet [28,42,43]. Magnetic properties of the obtained catalysts were elucidated with vibrating sample magnetometer (VSM) analyzer between the range of −20000 Oe +20000 Oe at room temperature. The magnetization saturation values (Ms) of MnFe_2_O_4_ is 52.12 emu g^−1^. However, the saturation magnetization of the silica-coated and Ag loaded MnFe_2_O_4_@SiO_2_-Ag NPs decreases as the silica shell thickness increases, and it has value of ~33.51 emu g^−1^ with shell thickness of 20 nm, respectively (Figure 3c).

ATR-FTIR spectroscopy was also used to monitor the SiO_2_ coating process of the MnFe_2_O_4_ surface and the Ag doping process with green synthesis [34,44]. The ATR-FTIR spectrum of MnFe_2_O_4_@SiO_2_ exhibits a broad band in the region 3400 cm^−1^ and fewer intense band at 1650 cm^−1^, which are due to O-H stretching and O-H deformation vibrations of coordinated water, respectively (Figure 3d) [45]. These O-H bands also include Si-OH stretchings and vibrations of SiO_2_. The bands centered at 1090 cm^−1^ and 810 cm^−1^ are, respectively, assigned to the vibrations of Si-O-Si (asym) and the vibration of Si-O-Si (sym) [46]. No significant change was observed in the ATR-FTIR spectra of MnFe_2_O_4_@SiO_2_-Ag on doping with AgNPs except minor intensity and position changes in the ~750–1250 cm^−1^ region. These results show that Ag nanoparticles formed by reduction with green tea extract do not cause deformation on the SiO_2_ surface.

### 3.2. Catalytic properties of MnFe_2_O_4_@SiO_2_-Ag

Over the last decade, industrial effluents bearing organic dye pollutants and stemming from various activities such as textile, plastic, cosmetic and have come to a serious problem to be overcome [47]. Due to their water solubility to some extend up to 10–200 mg/L, dye contaminants are regarded as one of the most important resources of the water pollution all over the world [48]. In spite of numerous methods, involving precipitation, adsorption or biogenic treatment have been employed; the concerns still maintain due to their high cost, generation of inadmissible side products that might lead to damages on animal and human, comprising of liver, kidney, etc. [47], and requisition of possible high-energy demands, especially in massive treatments [49]. Therefore, the complete removal of the organic pollutants from the industrial effluents by direct catalytic reductions has been occurring as a major environmentally friendly remedy [50].

**Figure 4:**
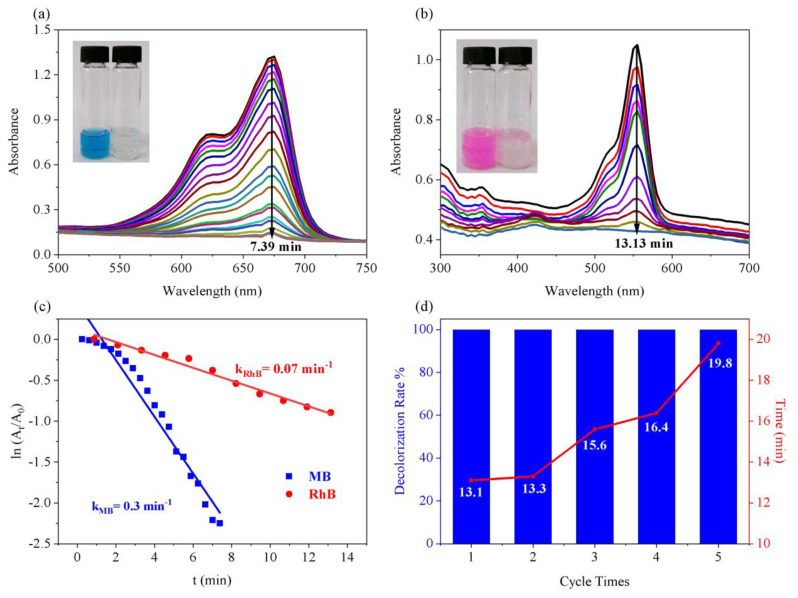
The reduction of MB (a) and RhB (b) in aqueous solution using MnFe_2_O_4_@SiO_2_-Ag nanocatalyst. The comparison of the first-order kinetic plots of MB and RhB in the presence of MnFe_2_O_4_@SiO_2_-Ag (c). Recycling of the MnFe_2_O_4_@SiO_2_-Ag for the reduction of RhB by NaBH_4_ (d).

**Scheme 2:**
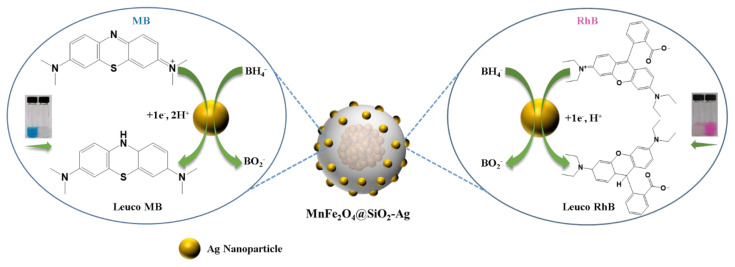
Possible mechanism of the reduction of MB and RhB catalyzed by MnFe_2_O_4_@SiO_2_-Ag.

Former studies have shown that AgNPs exhibited good catalytic activity and selectivity for various reactions [40,51,52]. In the present study, the catalytic performance of the green synthesized MnFe_2_O_4_@SiO_2_-Ag nanocatalyst by using *EP* extract was evaluated in the model direct reduction reactions of MB and RhB by NaBH_4_, as they are good representative members of the hazardous organic pollutants [53,54]. In addition, their decolorization processes can be easily monitored by naked eye and UV-Vis spectroscopy from the unique absorption bands at around 554 and 670 nm for RhB and MB, respectively [55,56]. Thus, the practical investigation of the degradation of MB and RhB could be beneficial for the purification of dye effluents. As it can be observed from Figures 4a and 4b, conversions of dyes were completed in 7.39 min (MB) and 13.13 min (RhB) after addition of the MnFe_2_O_4_@SiO_2_-Ag nanocatalyst to the individual solutions, including the excess amount of NaBH_4_. The color bleaching of the aqueous solutions together with the leveling off the UV-Vis bands after gradual decreases were also indicated the completion of the reduction reactions successfully. These results confirmed the successful degradation of MB and RhB to their leuco forms [34,57–60] by means of the redox reactions appearing on the surface of the electron relay systems (AgNPs) enabling the transfer of surface hydride ion electrons from BH_4_^−^ to the target acceptor dyes MB and RhB [48,60,61]. Possible reduction mechanism of the MB and RhB by MnFe_2_O_4_@SiO_2_-Ag was illustrated in Scheme 2. Taken into consideration the above results, it can be concluded that chromophore functional groups of C=N− and −N=N− present in MB and RhB have been successfully reduced to those of colorless C-N and N-N in the presence of immobilized AgNPs on the MnFe_2_O_4_@SiO_2_ surface [62,63].

In order to enlighten the catalytic role of the as synthesized nanocatalyst on diverse organic pollutants, rate constants for the MB and RhB reduction reactions were calculated and compared in Figure 4c. During the catalytic reduction studies, the concentration of the NaBH_4_ was used as excessively higher than the used dyes in order to obey the pseudo first-order kinetics described by *ln (A_t_ /A*_0_*) =* − *kt*, where *k, t*, *A_t_*, and *A*_0_ correspond to apparent rate constant, reaction time, absorbances of dyes at time “t” and “0”, respectively [64]. The obtained results revealed that the MnFe_2_O_4_@SiO_2_-Ag exhibited higher catalytic towards MB (0.3 min^−1^) than RhB (0.07 min^−1^) (Figure 4c). To further get a better insight into the catalytic activity of the MnFe_2_O_4_@SiO_2_-Ag and show the facility of this work, normalized rate constants (*k_nor_*=k/m, where the m is the catalyst mass) were calculated [65], and the performance of our catalyst was compared with the other catalyst systems in the literature. The results were summarized in Table. Compared with the other various metal-based catalyst systems, the catalytic activity of the green synthesized MnFe_2_O_4_@SiO_2_-Ag was distinctive and even satisfactory with the *k_nor_* values of 116.28 s^−1^ g^−1^ and 27.13 s^−1^ g^−1^ for MB and RhB, respectively. Therefore, it could be inferred that the MnFe_2_O_4_@SiO_2_-Ag nanocatalyst can be utilized with a good potential for the reduction of dye contaminants in water and be promising to future studies with its environmentally friendly preparation process by *EP* extract without using extra reducing or stabilizing agent that might be toxic for the living organism and the environment.

### 3.3. Recyclability of the MnFe_2_O_4_@SiO_2_-Ag

The recyclability and stability of the catalyst are important factors to show the sustainability of the core-shell magnetic nanocatalysts prepared by using *EP* extract for the immobilization of AgNPs on the MnFe_2_O_4_@SiO_2_ surface. Thus, the recyclability tests were conducted on the model reduction reaction of RhB by NaBH_4_ in the presence of the MnFe_2_O_4_@SiO_2_-Ag, and the obtained results were presented in Figure 4d. For a routine cyclic test, an external niobium magnet was used to separate the used nanocatalyst from the reaction media after the catalytic degradation. Before starting the subsequent cycle, the recycled nanocatalyst was washed several times with water and subsequently three times with ethanol. After dried under vacuum, they were used for the next cycle. In each cycle, the same procedure was repeated. Figure 4d shows the recyclability test results. In the formation of these graphs, the maximum absorbance values of the RhB were used to calculate percent decolorization rates. This overlapped plot drawn from the decolorization rate % and time (min) proves that the MnFe_2_O_4_@SiO_2_-Ag maintains its catalytic activity through the five repeated cycles without any loss in its decolorization efficiency (100%), so that the possibility of leaching AgNPs from the MnFe_2_O_4_@SiO_2_-Ag nanocomposites were ignored. Nevertheless, it can be also seen from Fig 4d that time required to complete the reaction in each cycle increases from 13.1 min (first cycle) to 19.8 min (fifth cycle). This increase could be attributed to loss of magnetic catalyst during the recovery process of the catalyst [65]. Overall, the produced MnFe_2_O_4_@SiO_2_-Ag coreshell magnetic nanocatalyst were stable and sufficient enough for the reduction of RhB, and they could be good candidates and have a great potential for the removal of dye contaminants in water.

## 4. Conclusion

In the current study, we showed a green synthetic strategy for the immobilization of AgNPs on manganese ferrite nanoparticles coated with the protective silica layer by using *EP* extract without facilitating any other reducing or stabilizing agents. This approach presents significant advantages over the existing ones in terms of using mild reaction conditions, requiring no extra reducing agent or surfactant, organic solvent, and hazardous materials. Bio-based process used here does not generate environmentally hazardous waste. For this reason, the reaction product occurring in these processes do not frequently need purification. The prepared catalyst system in this study revealed sufficient catalytic activity for the removal of MB and RhB compared with the previous studies. Moreover, the superior magnetization characteristics of the MnFe_2_O_4_@SiO_2_-Ag led them to be used several times without losing a prominent catalytic activity in each successive cycle. Thus, the obtained overall results suggest that the MnFe_2_O_4_@SiO_2_-Ag core-shell magnetic nanocomposites could be highly efficient and stable catalytic systems for the treatment of organic or dye contaminants and numerous applications in heterogeneous catalysis considering the environmental pollution concerns.

## Figures and Tables

**Table:** Comparison of the catalytic performances of MnFe_2_O_4_@SiO_2_-Ag with other catalyst system over MB and 4-NP reduction by NaBH_4_.

Dyes	Catalyst system	Catalyst mass (mg)	k (10^−3^ s^−1^)	k_app_ (s^−1^ g^−1^)	Time (min)	Ref.
MB	AgNPs	0.5	5.75	11.50	12	[66]
	Fe_3_O_4_@Ag	1.6	6.83	4.27	6	[67]
	MGO-PDA@Ag	3.0	7.12	2.37	7	[68]
	Ag/PSNM-3	2.0	2.23	1.12	11	[69]
	Fe_3_O_4_@HA@Ag	1	1.33	1.33	20	[70]
	Fe_3_O_4_@His@Ag	1	4.50	4.50	4	[71]
	MnFe_2_O_4_@SiO_2_-Ag	0.043	5.00	116.28	7.39	This work
RhB	Ag/TP	10	5.68	0.57	6	[72]
	Fe_3_O_4_@EDTA-Ag	30	34.00	1.13	3	[73]
	Fe_3_O_4_@Nico-Ag	1	3.83	3.83	10	[74]
	MnFe_2_O_4_@EP@Ag	0.0214	7.50	350.47	7.63	[25]
	AgCl@TA5.0-cellulose hydrogels	1	32.30	32.30	3	[75]
	MnFe_2_O_4_@SiO_2_-Ag	0.043	1.17	27.13	13.13	This work

## Data Availability

The datasets used and/or analyzed during the current study are available from the corresponding author on reasonable request.

## References

[1] Mani S Chowdhary P Bharagava RN Textile wastewater dyes: Toxicity profile and treatment approaches Emerging and eco-friendly approaches for waste management 2018 219 244 10.1007/978-981-10-8669-4_11

[2] Mondal P Baksi S Bose D Study of Environmental Issues in Textile Industries and Recent Wastewater Treatment Technology World Scientific News 2017 61 98 109

[3] Crini G Studies on adsorption of dyes on beta-cyclodextrin polymer Bioresource Technology 2003 90 2 193 198 10.1016/S0960-8524(03)00111-1 12895563

[4] Wijetunga S Li X-F Jian C Effect of organic load on decolourization of textile wastewater containing acid dyes in upflow anaerobic sludge blanket reactor Journal of Hazardous Materials 2010 177 1–3 792 798 10.1016/j.jhazmat.2009.12.103 20074855

[5] Kavimani T Senthilkumar PL Anaerobic Treatment of Dye Wastewater using Upflow Anaerobic Sludge Blanket Reactor International Journal of Innovative Technology and Exploring Engineering 2019 8 12 3178 3181 10.35940/ijitee.L3044.1081219

[6] Ertürk AS PAMAM dendrimer-enhanced removal of cobalt ions based on multiple-response optimization using response surface methodology Journal of the Iranian Chemical Society 2018 15 8 1685 1698 10.1007/s13738-018-1366-3

[7] Ambashta RD Sillanpää M Water purification using magnetic assistance: A review Journal of Hazardous Materials 2010 180 1–3 38 49 10.1016/j.jhazmat.2010.04.105 20488616

[8] Chan SHS Yeong Wu T Juan JC Teh CY Recent developments of metal oxide semiconductors as photocatalysts in advanced oxidation processes (AOPs) for treatment of dye waste-water Journal of Chemical Technology & Biotechnology 2011 86 9 1130 1158 10.1002/jctb.2636

[9] Burakov AE Galunin EV Burakova IV Kucherova AE Agarwal S Adsorption of heavy metals on conventional and nanostructured materials for wastewater treatment purposes: A review Ecotoxicology and Environmental Safety 2018 148 702 712 10.1016/j.ecoenv.2017.11.034 29174989

[10] Jana NR Wang ZL Pal T Redox Catalytic Properties of Palladium Nanoparticles: Surfactant and Electron Donor–Acceptor Effects Langmuir 2000 16 6 2457 2463 10.1021/la990507r

[11] Jana NR Pal T Redox Catalytic Property of Still-Growing and Final Palladium Particles: A Comparative Study Langmuir 1999 15 10 3458 3463 10.1021/la981512i

[12] Shimizu K Sawabe K Satsuma A Unique catalytic features of Ag nanoclusters for selective NOx reduction and green chemical reactions Catalysis Science & Technology 2011 1 3 331 341 10.1039/c0cy00077a

[13] Holmes AB Gu FX Emerging nanomaterials for the application of selenium removal for wastewater treatment Environmental Science: Nano 2016 3 5 982 996 10.1039/C6EN00144K

[14] Nasrollahzadeh M Mohammad Sajadi S Rostami-Vartooni A Khalaj M Green synthesis of Pd/Fe 3 O 4 nanoparticles using Euphorbia condylocarpa M. bieb root extract and their catalytic applications as magnetically recoverable and stable recyclable catalysts for the phosphine-free Sonogashira and Suzuki coupling reactions Journal of Molecular Catalysis A: Chemical 2015 396 31 39 10.1016/j.molcata.2014.09.029

[15] Veisi H Gholami J Ueda H Mohammadi P Noroozi M Magnetically palladium catalyst stabilized by diaminoglyoxime-functionalized magnetic Fe_3_O_4_ nanoparticles as active and reusable catalyst for Suzuki coupling reactions Journal of Molecular Catalysis A: Chemical 2015 396 216 223 10.1016/j.molcata.2014.10.012

[16] Baig RBN Varma RS Magnetically retrievable catalysts for organic synthesis Chemical Communications 2013 49 752 770 10.1039/C2CC35663E 23212208

[17] Ertürk AS Elmacı G PAMAM Dendrimer Functionalized Manganese Ferrite Magnetic Nanoparticles: Microwave-Assisted Synthesis and Characterization Journal of Inorganic and Organometallic Polymers and Materials 2018 28 5 2100 2107 10.1007/s10904-018-0865-0

[18] Bonyasi F Hekmati M Veisi H Preparation of core/shell nanostructure Fe3O4@PEG400-SO3H as heterogeneous and magnetically recyclable nanocatalyst for one-pot synthesis of substituted pyrroles by Paal-Knorr reaction at room temperature Journal of Colloid and Interface Science 2017 496 177 187 10.1016/j.jcis.2017.02.023 28219034

[19] Deng Y-H Wang C-C Hu J-H Yang W-L Fu S-K Investigation of formation of silica-coated magnetite nanoparticles via sol–gel approach Colloids and Surfaces A: Physicochemical and Engineering Aspects 2005 262 1–3 87 93 10.1016/j.colsurfa.2005.04.009

[20] Mohammadi P Sheibani H Green synthesis of Fe3O4@SiO2 -Ag magnetic nanocatalyst using safflower extract and its application as recoverable catalyst for reduction of dye pollutants in water Applied Organometallic Chemistry 2018 32 4 e4249 10.1002/aoc.4249

[21] Balentine DA Wiseman SA Bouwens LCM The chemistry of tea flavonoids Critical Reviews in Food Science and Nutrition 1997 37 8 693 704 10.1080/10408399709527797 9447270

[22] Graham HN Green tea composition, consumption, and polyphenol chemistry Preventive Medicine 1992 21 3 334 350 10.1016/0091-7435(92)90041-F 1614995

[23] Veisi H Ghorbani F Iron oxide nanoparticles coated with green tea extract as a novel magnetite reductant and stabilizer sorbent for silver ions: Synthetic application of Fe3O4@green tea/Ag nanoparticles as magnetically separable and reusable nanocatalyst for reduction of 4-nitrophenol Applied Organometallic Chemistry 2017 31 10 e3711 10.1002/aoc.3711

[24] Ertürk AS Controlled Production of Monodisperse Plant-Mediated AgNP Catalysts Using Microwave Chemistry: A Desirability-Function-Based Multiple-Response Optimization Approach ChemistrySelect 2019 4 32 9300 9308 10.1002/slct.201902197

[25] Gürbüz MU Koca M Elmacı G Ertürk AS In situ green synthesis of MnFe2O4@EP@Ag nanocomposites using Epilobium parviflorum green tea extract: An efficient magnetically recyclable catalyst for the reduction of hazardous organic dyes Applied Organometallic Chemistry 2021 35 6 e6230 10.1002/aoc.6230

[26] Ertürk AS Biosynthesis of Silver Nanoparticles Using Epilobium parviflorum Green Tea Extract: Analytical Applications to Colorimetric Detection of Hg2+ Ions and Reduction of Hazardous Organic Dyes Journal Cluster Science 2019 30 5 1363 1373 10.1007/s10876-019-01634-4

[27] Maklakov SS Lagarkov AN Maklakov SA Adamovich YA Petrov DA Corrosion-resistive magnetic powder Fe@SiO2 for microwave applications Journal of alloys and compounds 2017 706 267 273 10.1016/j.jallcom.2017.02.250

[28] Kayili HM Ertürk AS Elmacı G Salih B Poly(amidoamine) dendrimer-coated magnetic nanoparticles for the fast purification and selective enrichment of glycopeptides and glycans Journal of Separation Science 2019 42 20 3209 3216 10.1002/jssc.201900492 31389124

[29] Elmaci G Frey CE Kurz P Zümreoğlu-Karan B Water oxidation catalysis by using nano-manganese ferrite supported 1D-(tunnelled), 2D-(layered) and 3D-(spinel) manganese oxides Journal of Materials Chemistry A 2016 4 22 8812 8821 10.1039/c6ta00593d

[30] De G Karmakar B Ganguli D Hydrolysis-condensation reactions of TEOS in the presence of acetic acid leading to the generation of glass-like silica microspheres in solution at room temperature Journal of Materials Chemistry 2000 10 10 2289 2293 10.1039/b003221m

[31] Karmakar B De G Ganguli D Dense silica microspheres from organic and inorganic acid hydrolysis of TEOS Journal of Non-crystalline Solids 2000 272 2–3 119 126 10.1016/S0022-3093(00)00231-3

[32] Yang J Noble metal-based nanocomposites: Preparation and applications Weinheim, Germany John Wiley & Sons 2019 10.1002/9783527814305

[33] Gawande MB Goswami A Asefa T Guo H Biradar AV Core-shell nanoparticles: synthesis and applications in catalysis and electrocatalysis Chemical Society Reviews 2015 44 21 7540 7590 10.1039/c5cs00343a 26288197

[34] Kurtan U Amir M Yildiz A Baykal A Synthesis of magnetically recyclable MnFe2O4 @SiO 2 @Ag nanocatalyst: Its high catalytic performances for azo dyes and nitro compounds reduction Applied Surface Science 2016 376 16 25 10.1016/j.apsusc.2016.02.120

[35] Meral K Metin Ö Graphene oxide{magnetite nanocomposite as an effcient and magnetically separable adsorbent for methylene blue removal from aqueous solution Turkish Journal of Chemistry 2014 38 5 775 782 10.3906/kim-1312-28

[36] Rahimi R Tadjarodi A Imani M Rabbani M Moghaddam SS Kerdari H Synthesis of tetrakis(carboxyphenyl)porphyrin coated paramagnetic iron oxide nanoparticles via amino acid for photodegradation of methylene blue Turkish Journal of Chemistry 2013 37 6 879 888 10.3906/kim-1204-19

[37] Ahankar H Ramazani A Slepokura K Lis T Joo SW One-pot synthesis of substituted 4H-chromenes by nickel ferrite nanoparticles as an efficient and magnetically reusable catalyst Turkish Journal of Chemistry 2018 42 3 719 734 10.3906/kim-1710-14

[38] Hassani A Eghbali P Ekicibil A Metin Ö Monodisperse cobalt ferrite nanoparticles assembled on mesoporous graphitic carbon nitride (CoFe2O4/mpg-C3N4): A magnetically recoverable nanocomposite for the photocatalytic degradation of organic dyes Journal of Magnetism and Magnetic Materials 2018 456 400 412 10.1016/j.jmmm.2018.02.067

[39] Hassani A Çelikdağ G Eghbali P Sevim M Karaca S Heterogeneous sono-Fenton-like process using magnetic cobalt ferrite-reduced graphene oxide (CoFe2O4-rGO) nanocomposite for the removal of organic dyes from aqueous solution Ultrasonics Sonochemistry 2018 40 841 852 10.1016/j.ultsonch.2017.08.026 28946495

[40] Xie Y Yan B Xu H Chen J Liu Q Highly regenerable mussel-inspired Fe3O 4@Polydopamine-Ag core-shell microspheres as catalyst and adsorbent for methylene blue removal ACS Appl Mater Interfaces 2014 6 8845 8852 10.1021/am501632f 24787615

[41] Elmacı G Magnetic Hollow Biocomposites Prepared from Lycopodium clavatum Pollens as Efficient Recyclable Catalyst ChemistrySelect 2020 5 7 2225 2231 10.1002/slct.201904152

[42] Elmacı G Frey CE Kurz P Zümreoğlu-Karan B Water oxidation catalysis by birnessite@iron oxide core-shell nanocomposites Inorganic Chemistry 2015 54 6 2734 2741 10.1021/ic502908w 25710557

[43] Elmacı G Microwave-assisted rapid synthesis of C@Fe3O4 composite for removal of microplastics from drinking water Adıyaman University Journal of Science 2020 10 1 207 217 10.37094/adyujsci.739599

[44] Fan R Min H Hong X Yi Q Liu W Zhang Q Plant tannin immobilized Fe3O4@SiO2 microspheres: A novel and green magnetic bio-sorbent with superior adsorption capacities for gold and palladium Journal of Hazardous Materials 2019 364 780 790 10.1016/j.jhazmat.2018.05.061 30447562

[45] Dippong T Levei EA Goga F Cadar O Influence of Mn2+ substitution with Co2+ on structural, morphological and coloristic properties of MnFe2O4/SiO2 nanocomposites Materials Characterization 2021 172 110835 10.1016/j.matchar.2020.110835

[46] Dippong T Levei EA Lengauer CL Daniel A Toloman D Investigation of thermal, structural, morphological and photocatalytic properties of CuxCo1-xFe2O4 (0 ≤ x ≤ 1) nanoparticles embedded in SiO2 matrix Materials Characterization 2020 163 110268 10.1016/j.matchar.2020.110268

[47] Nadaf NY Kanase SS Biosynthesis of gold nanoparticles by Bacillus marisflavi and its potential in catalytic dye degradation Arabian Journal of Chemistry 2019 12 8 4806 4814 10.1016/j.arabjc.2016.09.020

[48] Jyoti K Singh A Green synthesis of nanostructured silver particles and their catalytic application in dye degradation Journal of Genetic Engineering and Biotechnology 2016 14 2 311 317 10.1016/j.jgeb.2016.09.005 30647629 PMC6299851

[49] Veisi H Azizi S Mohammadi P Green synthesis of the silver nanoparticles mediated by Thymbra spicata extract and its application as a heterogeneous and recyclable nanocatalyst for catalytic reduction of a variety of dyes in water Journal of Cleaner Production 2018 170 1536 1543 10.1016/j.jclepro.2017.09.265

[50] Gao P Tian X Yang C Zhou Z Li Y Wang Y Fabrication, performance and mechanism of MgO meso-/macroporous nanostructures for simultaneous removal of As( III ) and F in a groundwater system Environmental Science: Nano 2016 3 6 1416 1424 10.1039/C6EN00400H

[51] Kaloti M Kumar A Sustainable Catalytic Activity of Ag-Coated Chitosan-Capped γ-Fe 2 O 3 Superparamagnetic Binary Nanohybrids (Ag-γ-Fe2O3@CS) for the Reduction of Environmentally Hazardous Dyes-A Kinetic Study of the Operating Mechanism Analyzing Methyl Orange Reductio ACS Omega 2018 3 2 1529 1545 10.1021/acsomega.7b01498 31458478 PMC6641453

[52] Cheng XQ Wang ZX Guo J Ma J Shao L Designing Multifunctional Coatings for Cost-Effectively Sustainable Water Remediation ACS Sustainable Chemistry & Engineering 2018 6 2 1881 1890 10.1021/acssuschemeng.7b03296

[53] Das R Sypu VS Paumo HK Bhaumik M Maharaj V Maity A Silver decorated magnetic nanocomposite (Fe3O4@PPy-MAA/Ag) as highly active catalyst towards reduction of 4-nitrophenol and toxic organic dyes Applied Catalysis B: Environmental 2019 244 546 558 10.1016/j.apcatb.2018.11.073

[54] Abay AK Chen X Kuo D-H Highly efficient noble metal free copper nickel oxysulfide nanoparticles for catalytic reduction of 4-nitrophenol, methyl blue, and rhodamine-B organic pollutants New Journal Chemistry 2017 41 13 5628 5638 10.1039/C7NJ00676D

[55] Xu Y Shi X Hua R Zhang R Yao Y Remarkably catalytic activity in reduction of 4-nitrophenol and methylene blue by Fe3O4@COF supported noble metal nanoparticles Applied Catalysis B: Environmental 2020 260 118142 10.1016/j.apcatb.2019.118142

[56] Xu P Cen C Zheng M Wang Y Wu Z A facile electrostatic droplets assisted synthesis of copper nanoparticles embedded magnetic carbon microspheres for highly effective catalytic reduction of 4-nitrophenol and Rhodamine B Materials Chemistry and Physics 2020 253 123444 10.1016/j.matchemphys.2020.123444

[57] Cui K Yan B Xie Y Qian H Wang X Regenerable urchin-like Fe3O4 @PDA-Ag hollow microspheres as catalyst and adsorbent for enhanced removal of organic dyes Journal of Hazardous Materials 2018 350 66 75 10.1016/j.jhazmat.2018.02.011 29453121

[58] Yang Y Ji H Duan H Fu Y Xia S Controllable synthesis of mussel-inspired catechol-formaldehyde resin microspheres and their silver-based nanohybrids for catalytic and antibacterial applications Polymer Chemistry 2019 10 33 4537 4550 10.1039/C9PY00846B

[59] Sun L He J An S Zhang J Zheng J Recyclable Fe3O4@SiO2-Ag magnetic nanospheres for the rapid decolorizing of dye pollutants Chinese Journal of Catalysis 2013 34 7 1378 1385 10.1016/s1872-2067(12)60605-6

[60] Veisi H Razeghi S Mohammadi P Hemmati S Silver nanoparticles decorated on thiol-modified magnetite nanoparticles (Fe3O4/SiO2 - Pr-S-Ag) as a recyclable nanocatalyst for degradation of organic dyes Materials Science and Engineering: C 2019 97 624 631 10.1016/j.msec.2018.12.076 30678949

[61] Wang Y Gao P Wei Y Jin Y Sun S Silver nanoparticles decorated magnetic polymer composites (Fe3O4@PS@Ag) as highly efficient reusable catalyst for the degradation of 4-nitrophenol and organic dyes Journal of Environmental Management 2021 278 111473 10.1016/j.jenvman.2020.111473 33120097

[62] Gürbüz MU Ertürk AS Synthesis and Characterization of Jeffamine Core PAMAM Dendrimer-Silver Nanocomposites (Ag JCPDNCs) and Their Evaluation in The Reduction of 4-Nitrophenol Journal of the Turkish Chemical Society Section A: Chemistry 2018 5 2 885 894 10.18596/jotcsa.428572

[63] Corma A Concepción P Serna P A Different Reaction Pathway for the Reduction of Aromatic Nitro Compounds on Gold Catalysts Angewandte Chemie 2007 46 41 7820 7822 10.1002/ange.200700823 17579907

[64] Zhang J Fang Q Duan J Xu H Xu H Magnetically Separable Nanocatalyst with the Fe3O4 Core and Polydopamine-Sandwiched Au Nanocrystal Shell Langmuir 2018 34 14 4298 306 10.1021/acs.langmuir.8b00302 29546989

[65] Qin L Huang D Xu P Zeng G Lai C In-situ deposition of gold nanoparticles onto polydopamine-decorated g-C3N4 for highly efficient reduction of nitroaromatics in environmental water purification Journal of Colloid and Interface Science 2019 534 357 369 10.1016/j.jcis.2018.09.051 30243177

[66] Ajitha B Reddy YAK Lee Y Kim MJ Ahn CW Biomimetic synthesis of silver nanoparticles using Syzygium aromaticum (clove) extract: Catalytic and antimicrobial effects Applied Organometallic Chemistry 2019 33 5 e4867 10.1002/aoc.4867

[67] Sharma G Jeevanandam P A facile synthesis of multifunctional iron oxide@Ag core-shell nanoparticles and their catalytic applications European Journal of Inorganic Chemistry 2013 6126 6136 10.1002/ejic.201301193

[68] Upoma BP Mahnaz F Rahman Sajal W Zahan N Hossain Firoz MS Bio-inspired immobilization of silver and gold on magnetic graphene oxide for rapid catalysis and recyclability Journal of Environmental Chemical Engineering 2020 8 3 103739 10.1016/j.jece.2020.103739

[69] Liao G Li Q Zhao W Pang Q Gao H In-situ construction of novel silver nanoparticle decorated polymeric spheres as highly active and stable catalysts for reduction of methylene blue dye Applied Catalysis A: General 2018 549 102 111 10.1016/j.apcata.2017.09.034

[70] Amir M Güner S Yıldız A Baykal A Magneto-optical and catalytic properties of Fe3O4@HA@Ag magnetic nanocomposite Journal of Magnetism and Magnetic Materials 2017 421 462 471 10.1016/j.jmmm.2016.08.037

[71] Amir M Kurtan U Baykal A Rapid color degradation of organic dyes by Fe3O4@His@Ag recyclable magnetic nanocatalyst Journal of Industrial and Engineering Chemistry 2015 27 347 353 10.1016/j.jiec.2015.01.013

[72] Ismail M Khan MI Khan SB Akhtar K Khan MA Catalytic reduction of picric acid, nitrophenols and organic azo dyes via green synthesized plant supported Ag nanoparticles Journal of Molecular Liquids 2018 268 87 101 10.1016/j.molliq.2018.07.030

[73] Sharif HMA Mahmood A Cheng H-Y Djellabi R Ali J Fe3O4 Nanoparticles Coated with EDTA and Ag Nanoparticles for the Catalytic Reduction of Organic Dyes from Wastewater ACS Applied Nano Materials 2019 2 8 5310 5319 10.1021/acsanm.9b01250

[74] Kurtan U Amir M Baykal A Fe3O4@Nico-Ag magnetically recyclable nanocatalyst for azo dyes reduction Applied Surface Science 2016 363 66 73 10.1016/j.apsusc.2015.11.214

[75] Zhang M Li M Yu N Su S Zhang X Fabrication of AgCl@tannic acid-cellulose hydrogels for NaBH4-mediated reduction of 4-nitrophenol Cellulose 2021 28 6 3515 3529 10.1007/s10570-021-03721-0

